# Isoflurane attenuates left ventricular akinesia and preserves cardiac output in the Tako-tsubo rat model

**DOI:** 10.1186/cc13377

**Published:** 2014-03-17

**Authors:** J Oras, B Redfors, Y Shao, H Seeman-Lodding, SE Ricksten, E Omerovic

**Affiliations:** 1 Institute of Clinical Sciences, Gothenburg, Sweden; 2 The Wallenberg Laboratory, Gothenburg, Sweden

## Introduction

Tako-tsubo cardiomyopathy (TCM) is an acute cardiac syndrome with regional hypokinesia in the left ventricle (LV), often affecting the apex causing apical ballooning. TCM is frequent in patients with adrenergic overstimulation and is probably common in ICU patients [1]. In a TCM rat model we evaluated whether different anesthetic agents could attenuate LV akinesia in TCM.

## Methods

Isoprenaline was intraperitoneal (i.p.) injected, which induces LV akinesia and apical ballooning within 90 minutes [2]. We performed the study in two different settings. In the first setting, spontaneously breathing rats (*n *= 12 in each group) were sedated with either pentobarbital, ketamine, isoflurane or no anesthetic before i.p. isoprenaline. One additional group received the K-ATP blocker glyburide before sedation with isoflurane. In the second setting, rats were anaesthetized with ketamine + midazolam, mechanically ventilated and the carotid artery was cannulated. Before i.p. isoprenaline, animals were randomized to either no isoflurane (0 MAC), isoflurane 0.5 MAC or isoflurane 1.0 MAC (*n *= 12 in each group). Arterial blood gas was obtained before isoprenaline and 60 minutes after isoprenaline. The heart rate (HR), systolic blood pressure (SBP) and body temperature (BT) were recorded continuously. After 90 minutes, echocardiography was performed. Extent of akinesia was expressed as the percentage of total LV endocardial length. End-diastolic and end-systolic LV volumes were measured, and stroke volume (SV) and cardiac output (CO) were calculated.

## Results

In spontaneously breathing rats, the degree of akinesia was significantly lower with pentobarbital and isoflurane (± glyburide) but not with ketamine compared with controls. The degree of akinesia was lowest with isoflurane. In ventilated rats, the degree of apical akinesia (%) was significantly lower at 0.5 MAC (8.7 ± 7.3) and 1 MAC (5.7 ± 7.4) versus 0 MAC (17.7 ± 8.0). This was accompanied by a higher CO and SV. HR was lower at 1 MAC (6%) and SBP was lower at 0.5 MAC (106 ± 7) and 1 MAC (98 ± 7) versus 0 MAC (126 ± 8). BT and pH was lower in both isoflurane groups. In a multivariate model, isoflurane was the only variable that was independently associated with the degree of LV akinesia.

## Conclusion

Isoflurane prevents experimental TCM and preserves LV function, an effect not mediated via opening of K-ATP channels. The effect cannot be explained entirely by attenuation of myocardial stress. Isoflurane sedation in the ICU might be an interesting approach for patients suffering from hyperadrenergic conditions at risk of developing TCM.
